# Effect of the LifeSpan suicide prevention model on self-harm and suicide in four communities in New South Wales, Australia: a stepped-wedge, cluster randomised controlled trial

**DOI:** 10.1136/bmjment-2024-301429

**Published:** 2025-03-31

**Authors:** Fiona Shand, Michelle Torok, Andrew Mackinnon, Alexander Burnett, Lisa N Sharwood, Philip J Batterham, Alison L Calear, Jiahui Qian, Stephanie Zeritis, Grant Sara, Andrew Page, Henry Cutler, Myfanwy Maple, Brian Draper, Lauren McGillivray, Matthew Phillips, Demee Rheinberger, Isabel Zbukvic, Helen Christensen

**Affiliations:** 1 Black Dog Institute, Randwick, New South Wales, Australia; 2 University of New South Wales, Sydney, New South Wales, Australia; 3 Australian National University, Canberra, Australian Capital Territory, Australia; 4 New South Wales Ministry of Health, St Leonards, New South Wales, Australia; 5 Western Sydney University, Penrith, New South Wales, Australia; 6 Macquarie University, Sydney, New South Wales, Australia; 7 University of New England, Armidale, New South Wales, Australia; 8 Orygen The National Centre of Excellence in Youth Mental Health, Parkville, Victoria, Australia; 9 University of Melbourne, Parkville, VIC, Australia

**Keywords:** Suicide & self-harm

## Abstract

**Background:**

There have been few rigorous evaluations of population, multi-strategy, suicide prevention programmes, despite increasing global recognition that such approaches are needed to reduce suicide.

**Objective:**

To examine the effects of a multi-strategy suicide prevention model on age-standardised rates of hospital presenting self-harm and suicide after 24 months of implementation.

**Methods:**

A stepped-wedge cluster randomised trial was conducted in four sites across New South Wales (NSW), Australia, from 2016 to 2020. Sites were randomised to a starting order and implemented the same set of interventions over a 24-month period. Changes in rates of hospital presenting self-harm and suicide deaths were measured using linked administrative health data sets of persons aged 10 or older.

**Results:**

Negative binomial regression models adjusted for linear trends and seasonality showed that LifeSpan was associated with a 13∙8% (incident response rate 0.86; 95% CI 0.79 to 0.94) reduction in hospital-presenting self-harm rates over the intervention period, compared with preintervention. These effects were not observed in the rest of NSW. There were statistically non-significant changes in suicide death rates during the intervention across all sites.

**Conclusions:**

Locally implementing a multi-strategy suicide prevention model can reduce rates of hospital presentations for self-harm, but longer implementation and evaluation periods may be required to realise the full impacts of interventions for suicide, as a more intractable outcome.

**Clinical implications:**

Our findings can inform policy at all levels of government to invest in actions that may build cross-sectoral capacity in local communities to detect and respond to suicide risk.

**WHAT IS ALREADY KNOWN ON THIS TOPIC**

- Multi-strategy prevention approaches have greater potential to reduce self-harm and suicide rates than standalone interventions, but there have only been a limited number of trials of such approaches to date, and findings have been equivocal.
- Methodological confounding related to the design and testing of prior models invites further investigation of whether multilevel approaches work, how and for what outcomes.

**WHAT THIS STUDY ADDS**

- This is the first stepped-wedge randomised controlled trial to causally establish short and medium-term effects of a multi-strategy suicide prevention model on self-harm and suicide rates.
- The LifeSpan multi-strategy suicide prevention model represents the first of its kind in Australia, and the most complex model of its kind, globally.

**HOW THIS STUDY MIGHT AFFECT RESEARCH, PRACTICE OR POLICY**

- Prioritising evidence-based strategies, multi-sectoral collaboration and local governance are important ingredients for multi-strategy suicide prevention approaches to work.
- Delivering a complex and multiyear suicide prevention model into local communities is a challenging but feasible and promising approach to reducing rates of hospital-presenting self-harm in the population.

## Background

 Intentional self-harm (ISH) contributes significantly to global death and disability, with many western countries, including Australia, showing no reduction in suicide death and attempt rates in the past two decades.^1 2^ Though WHO guidelines recommend the use of multiple evidence-based strategies, multisectoral collaboration and surveillance and monitoring for suicide prevention,^3^ only a handful of studies have trialled such models, providing equivocal evidence for their effectiveness. For example, multi-strategy suicide prevention trials in Germany, Munich, and Japan have demonstrated statistically significant reductions in suicidal behaviour at a whole-of-population level (7–24%) after 2 or more years of implementation,^4^,^6^ while other European trials have shown null intervention effects.^7 8^ In the case of New Zealand, a 7% increase in ISH rates was observed relative to control sites.^9^ While reasons for these mixed findings are unclear, ineffectuality may be attributable to difficulties implementing the model with the intended saturation and fidelity, inattention to the evidence base when selecting strategies^9^ and use of trial designs that do not consider effects of time and affect causal inference.^6^,^8^

The LifeSpan model was developed in accordance with the WHO’s best-practice recommendations in suicide prevention. The model involves the implementation of nine evidence-based universal, selective and indicated strategies^10 11^ across education, community and health settings, in defined geographical regions that represent communities, making it the most complex model of its kind. LifeSpan also had a stronger focus on building and supporting regional suicide prevention collaboratives to provide local governance relative to previous trials,^9^ in recognition that shared decision-making and local planning and action are facilitators of health promotion and prevention.^12^ A stepped-wedge (SW) randomised trial design was used to allow the LifeSpan model to ‘saturate’ the local regions in which it was implemented without sacrificing the causal testability of our primary hypothesis, that the age-standardised rate of suicide attempts, as indexed by hospital-treated ISH, would be lower after the 24 months of its implementation compared with preimplementation rates. The design also satisfied important ethical considerations, as it allowed exposure to the intervention for all sites, which were selected on the basis of high suicide rates. We also hypothesised that significant reductions in suicide rates would be observed at the end of the implementation period, and that the changes in ISH and suicide deaths across the trial sites would not be observed in the rest of the state.

## Methods

This study is reported as per the Consolidated Standards of Reporting Trials for SW trials (SW-cluster randomised trial)^13^ (online supplemental S1 checklist). The LifeSpan protocol was prospectively registered with the Australia New Zealand Clinical Trials Register (ACTRN12617000457347) and is published elsewhere.^14^

### Study design

The trial was conducted at four sites in New South Wales (NSW), Australia. An SW randomised trial design was used, involving the random and sequential crossover of the trial sites from control to intervention at stepped 4 month intervals between 1 August 2017 and 31 March 2018. The baseline period for sites varied between 67 and 75 months depending on the order of crossover, followed by a 24-month implementation phase and a postimplementation phase of up to 16 months (online supplemental figure S1). Linked hospital administrative data was used to measure ISH presentations and coronial data was used to assess suicide deaths.

### Sites

Each trial site comprised one or more NSW Local Government Areas falling within the boundaries of a single local health district or primary health network. Eligible sites had higher-than-state-average rates of suicide and/or hospital presentations for ISH, at least one emergency department, and a minimum estimated resident population of 145 000 to ensure sufficient power to detect a primary endpoint effect. The power calculation assumed up to 30% decrease in the proportion (rate) of ISH between any two sites with 90% power. Spatial modelling of suicide and ISH in NSW showed that 25 Local Government Areas across NSW met these criteria.^15^ Lead health agencies in these regions were invited to submit an expression of interest for site selection in April 2016. Applications were assessed against additional criteria, including implementation capacity, by a panel of five individuals holding senior positions in the mental health and suicide prevention sectors, who were not involved in the day-to-day activities of the trial.

### Randomisation

Sites were randomly allocated as clusters to the order in which they would crossover from the control to the intervention phase. Randomisation was conducted by an independent statistician. The ‘sample’ function in R (blockrand)^16^ was used to generate the random allocation sequence for the sites. Health agency leads in each trial site were informed of their allocated starting order and enrolled into the trial by the implementation manager (not part of the research team) prior to the intervention establishment phase. No additional randomisation was undertaken at the intervention level. Blinding at the site level was not possible due to the nature of the trial design, and as secondary data was used for evaluation, individual participant blinding was not applicable. The trial statistician was blinded to the order in which sites were allocated to crossover to minimise analysis bias.

### Procedure

We developed our LifeSpan multi-strategy suicide prevention model based on a review of evidence. Programmes were included that had Level 1 (systematic review of randomised controlled trials (RCTs)/single high-quality RCTs) or Level 2 (systematic review of cohort studies) evidence for reducing suicide attempts and/or suicide deaths, according to the Oxford Centre for Evidence Based Medicine grading scheme used by Mann and colleagues.^11^,^17^ 12 commercially available interventions were identified from this phase, which were fitted across nine strategy areas, spanning universal, selective, and indicated prevention and health promotion approaches (online supplemental S1 text). The implementation dosage targets for each intervention were established by a central research team at Black Dog Institute (BDI; online supplemental S1 text). Two local site coordinators per trial site were appointed to implement the model, supported by the BDI central team, a local governance committee (established for the trial) and their relevant regional suicide prevention collaborative, which included representatives from community, frontline, health and education organisations and services. The order of intervention delivery was not prescribed, but sites were expected to establish all interventions within the first 12 months of the implementation period.

### Outcomes

#### Intentional self-harm

The primary endpoint was change in the annual average rates of episodes of hospital-presenting ISH, measured using a probabilistic administrative linkage of hospital data sets. De-identified patient level data from the NSW Health Emergency Department Data Collection and NSW Admitted Patient Data Collection (APDC) were obtained from the NSW Centre for Health Record Linkage for the period of 1 January 2012 to 31 December 2020 and linked using the project-specific person number and episode start date time. ISH presentations were identified using International Classification of Diseases, 10th revision, Australian Modification (ICD-10-AM) codes (X60–X84, Y87.0) and a binary ISH variable (self-harm, yes/no) derived from an unstructured free text field relating to the presenting problem for the emergency department visit.^18^ Episodes of hospital-presenting ISH were geographically assigned using statistical areas Level 2 codes derived from the Australian Statistical Geographical Standard (Volume 1, 2016) and based on residential address. Monthly rates of ISH were calculated using interpolation of annual Australian Bureau of Statistics Estimated Residential Populations (ERPs) for the years 2012–2019. For the year 2020, the populations were extrapolated to provide monthly figures. These rates were age-standardised using Australian 2001 population estimates, calculated for each site and then reported as an aggregated rate change for all sites, combined and separately.

Though our published protocol paper stated that change in rates of suicide attempts would be our primary outcome,^13^ suicidal intent is not routinely captured by hospitals such that it was not possible to determine which self-harm presentations constituted a suicide attempt.

#### Suicide

Individual records were sourced from the cause of death unit record file (COD-URF) and Registry of Births and the Deaths and Marriages data sets, linked probabilistically. Records of confirmed suicides were available from 1 January 2012 to 31 December 2019. Due to lags in confirming suicide deaths, we do not have complete data for this outcome for the final 3 months of the trial, from 1 January to 31 March 2020. The COD was coded according to the ICD-10-AM (X60-X84, Y87.0) in COD-URF. For both ISH and suicide, records for children aged<10 years were excluded.

Due to low monthly suicide deaths (<20) at each individual site, we calculated quarterly crude rates using the same geographical allocation and ERP calculation methods that were used for determining ISH rates. Demographic characteristics included in the linked data sets were sex (female, male) and age in years.

### Sample size

Based on the NSW APDC, the mean incident rate of ISH hospital presentations in the period 2005–2013 was approximately 250 per 100 000 per year in the four target sites, combined. Using G*Power V.3.1,^19^ we used a conservative approach for our power analysis that considered changes in ISH incidence within each site, resulting in an estimate of 80% power to detect>20% decreases in each site, assuming population sizes of 145 000–190 000.

### Statistical analysis

**Figure 1:**
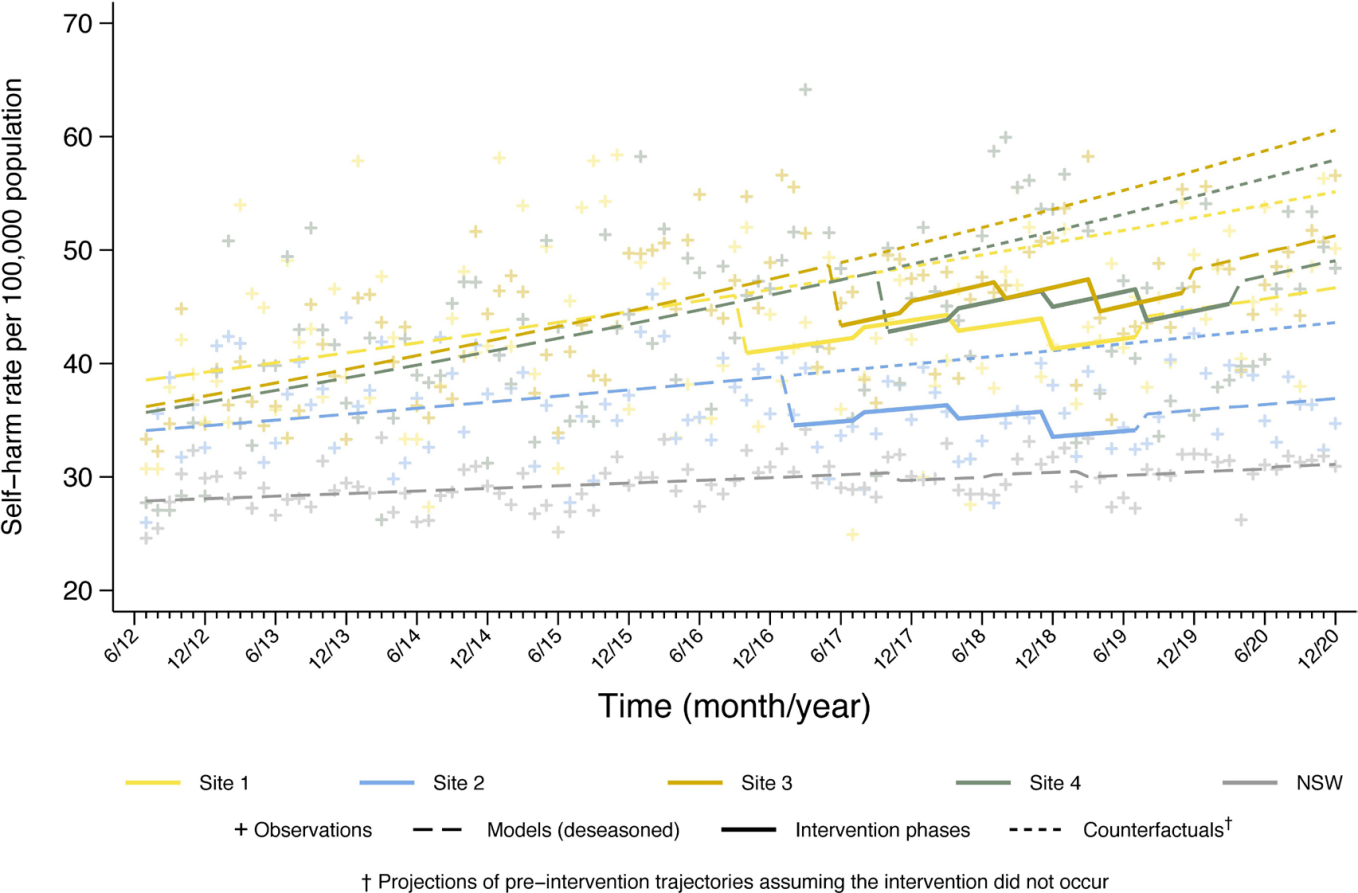
Changes over time in self-harm hospitalisation rates (per 100 000) by trial sites and across New South Wales (NSW).

A prespecified analysis plan governed our analyses,^14^ with the between-site comparisons and site-level sex differences conducted as post hoc analyses. For the primary hypothesis, negative binomial regression modelled rates of ISH as a function of time, season, site and intervention stage. Estimated resident population was included as an exposure variable. Linear slopes reflected long-term trends which, with intercepts, were permitted to differ between sites. A sinusoidal function common to all sites, with phase (month ‘zero’) chosen to maximise model likelihood and, common to all sites, accommodated seasonal variation. For analysis, the full trial period at each site was coded into the following time periods: preintervention (all data from July 2012 to the transition period), a 6-month transition period (excepting site 1 which had a 10-month transition period factored in to accommodate implementation delays), the 24-month implementation phase (which was divided into three 8-month periods designated as early, mid and late stage) and a postintervention phase (divided into two 8-month periods: post 1, post 2), although not all sites contributed complete data to the later stage due to the study design (figure 1). The postintervention period was included to evaluate longer-term impacts of the LifeSpan model. The seven stages were defined to allow patterns of establishment, stability and attenuation to be detected without imposing any form of such change on the outcome. The test of the primary hypothesis of effectiveness compared the average rates over early, mid and late stages of implementation to the preintervention rate in a model with intervention effects common to all sites. Subsequently, models were fitted to each site separately and to rest-of-state data for NSW (excluding trial sites). Average site times of commencement of intervention stages were used, with comparable models fitted to the NSW data. In exploratory analysis, rates were examined by sex for each site, for all sites combined, and for the rest of NSW.

Model evaluation included assessment of non-linear time trends and arbitrary patterns of seasonality. Maldistribution of residuals and the absence of influential outliers were investigated using the DHARMa (Diagnostics for Hierarchical Regression Models) package^20^ in R, which uses a simulation-based approach to create scaled residuals for fitted generalised linear models. As methods for multivariate time series are not well established, autocorrelation was investigated in models for individual sites. The Newey-West estimator^21^ was used with a range of lags to establish the general robustness of primary outcomes to autocorrelation.

For analysis of suicide deaths, similar methods were applied using negative binomial regression modelled rates of suicide death as a function of time, site and intervention stage. Quarterly crude rates of suicide death were calculated and the trial period at each site was coded into four time periods: preintervention (all data from July 2012 to transition), the transition period, ‘early’ implementation (first four quarters of implementation) and ‘late’ implementation (final four quarters of implementation). Data relating to the final quarter of implementation in site four and postintervention were not included for death analysis due to data unavailability.

Significance for both outcomes was set at p<0∙05 and is reported as incident rate ratios (IRR) with 95% CI. All analyses were undertaken using Stata V.18 and R V.4.2.1.

### Deviation from protocol

There was a deviation from the planned timing of crossover into the intervention phase for site 1, which was delayed due to unanticipated challenges in establishing the Swedish-regulated Youth Aware of Mental Health programme in schools. As a consequence, site 1 and site 2 both initiated their implementation phase in August 2017, and this has been accounted for in analyses.

## Results

### Intentional self-harm

We identified 14 022 individuals who presented to hospital for 22 387 episodes of ISH during the preintervention period. Baseline characteristics of hospital-presenting ISH are presented in table 1. The online supplemental figure S2 shows the number of records provided by the outlined sampling frame and from the discrete data sets held by NSW Health.

**Table 1:** Characteristics of trial sites, self-harm presentations and suicide deaths for the baseline (preintervention) period

	Site 1	Site 2	Site 3	Site 4	All sites
Site name	Newcastle	Illawarra Shoalhaven	Central Coast	Murrumbidgee	–
Lead agency	Hunter New England Local Health District	Coordinaire—Southeastern NSW Primary Health Network	Central Coast Local Health District	Murrumbidgee Primary Health Network	–
24-month implementation phase	1 August 2017 to 31 July 2019	1 August 2017 to 31 July 2019	1 December 2017 to 30 November 2019	1 April 2018 to 31 March 2020	–
Estimated residential population	171 316	393 204	327 736	164 364	1 056 620
Sex					
Female n (%)	86 857 (50.7)	199 748 (50.8)	169 112 (51.6)	82 675 (50.3)	538 392 (51.0)
Male n (%)	84 459 (49.3)	193 456 (49.2)	158 624 (48.4)	81 689 (49.7)	518 228 (49.0)
Geographical classification	Urban/coastal	Regional/coastal	Urban/coastal	Regional	–
No. hospitals with EDs	2	4	2	5	–
Self-harm					
Number of unique individuals presenting for self-harm	2414	4460	4820	2328	14 022
Intentional self-harm episodes n (%)	3951 (17.7)	7037 (31.4)	7306 (32.6)	4093 (18.3)	22 387
Age, mean (SD)	34.2 (17.2)	35.4 (18.8)	35.0 (19.1)	33.1 (17.6)	34.7 (18.5)
Sex					
Female n (%)	1342 (55.6)	2593 (58.1)	2787 (57.8)	1286 (55.2)	8008 (57.1)
Male n (%)	1072 (44.4)	1867 (41.9)	2033 (42.2)	1042 (44.8)	6014 (42.9)
Suicide deaths					
Number of deaths	104	177	214	130	625
Age, mean (SD)	44.1 (17.0)	45.9 (19.0)	48.2 (18.9)	45.1 (18.3)	46.5 (18.5)
Sex					
Female n (%)	27 (26.0)	50 (28.3)	57 (26.6)	19 (14.6)	153 (24.5)
Male n (%)	77 (74.0)	127 (71.8)	157 (73.4)	111 (85.4)	472 (75.5)

ED, emergency departmentNSW, New South Wales

Table 2 shows parameter estimates for the final model fitted across all sites. Corresponding observed and predicted ISH rates are graphed in figure 1. Adjusting for linear trend and seasonality, the IRRs at different intervention stages indicate reductions in rates of ISH compared with pre-intervention of 9.7% early in the intervention, increasing to 18.5% in the late intervention phase. Over the whole intervention period, LifeSpan was associated with a decrease in ISH rates of 13.8% compared with the preintervention period (IRR 0.86, 95% CI 0.79 to 0.94, p<0.001). The reductions in ISH observed in the intervention sites were not seen in the rest of NSW, where deviations from long-term trends during the intervention period in LifeSpan were negligible and statistically non-significant at all stages.

**Table 2:** Model parameter estimates for intentional self-harm (ISH) expressed as incident rates (per 100 000) and incident rate ratios for the model fitted to all sites and by sex, to each site separately and to the rest of NSW (95% CIs)

	All sites	Sex		Individual sites				Rest of NSW
		Males	Females	Site 1	Site 2	Site 3	Site 4	
Intercept								
Site 1	38.405***	32.666***	44.349***	38.900***	—	—	—	—
	(35.216 to 41.884)	(29.977 to 35.597)	(41.131 to 47.819)	(34.190 to 44.259)				
Site 2	34.004***	25.880***	42.084***	—	35.215***	—	—	—
	(31.352 to 36.880)	(24.085 to 27.808)	(39.489 to 44.849)		(31.945 to 38.820)			
Site 3	36.015***	30.124***	41.996***	—	—	35.223***	—	—
	(33.178 to 39.096)	(28.028 to 32.377)	(39.336 to 44.836)			(32.789 to 37.838)		
Site 4	35.522***	28.919***	42.274***	—	—	—	34.977***	—
	(32.506 to 38.819)	(26.422 to 31.652)	(39.086 to 45.722)				(31.081 to 39.361)	
Rest of NSW	—	—	—	—	—	—	—	27.852***
								(25.860 to 29.997)
Slope								
Site 1	1.004***	1.001	1.005***	1.003	—	—	—	—
	(1.002 to 1.006)	(0.999 to 1.003)	(1.003 to 1.007)	(0.999 to 1.008)				
Site 2	1.002*	1.003**	1.002**	—	1.001	—	—	—
	(1.001 to 1.004)	(1.001 to 1.005)	(1.001 to 1.004)		(0.998 to 1.004)			
Site 3	1.005***	1.006***	1.005***	—	—	1.006***	—	—
	(1.003 to 1.007)	(1.004 to 1.007)	(1.003 to 1.006)			(1.004 to 1.008)		
Site 4	1.005***	1.003***	1.006***	—	—	—	1.005**	—
	(1.003 to 1.007)	(1.001 to 1.005)	(1.004 to 1.007)				(1.002 to 1.008)	
NSW	—	—	—	—	—	—	—	1.001
								(0.999 to 1.004)
Seasonality								
Month	1.061***	1.073***	1.057***	1.077*	1.073**	1.066**	1.044	1.051*
	(1.033 to 1.090)	(1.048 to 1.100)	(1.034 to 1.080)	(1.008 to 1.151)	(1.018 to 1.131)	(1.026 to 1.107)	(0.977 to 1.117)	(1.009 to 1.095)
Evaluation period								
Pre	1	1	1	1	1	1	1	1
	—	—	—	—	—	—	—	—
Transition	0.886**	0.903*	0.879***	0.850	0.942	0.875*	0.918	1.001
	(0.809 to 0.970)	(0.831 to 0.981)	(0.816 to 0.946)	(0.693 to 1.042)	(0.786 to 1.130)	(0.768 to 0.996)	(0.733 to 1.149)	(0.875 to 1.144)
Early	0.903*	0.923	0.883**	0.803	0.922	0.852*	1.044	0.977
	(0.822 to 0.991)	(0.848 to 1.004)	(0.819 to 0.952)	(0.629 to 1.024)	(0.773 to 1.100)	(0.751 to 0.967)	(0.845 to 1.289)	(0.852 to 1.120)
Mid	0.871**	0.920	0.846***	0.858	0.902	0.882	0.855	0.984
	(0.787 to 0.965)	(0.840 to 1.008)	(0.779 to 0.918)	(0.658 to 1.119)	(0.743 to 1.095)	(0.770 to 1.010)	(0.680 to 1.075)	(0.848 to 1.141)
Late	0.815***	0.852**	0.797***	0.860	0.925	0.772***	0.734*	0.967
	(0.730 to 0.911)	(0.771 to 0.942)	(0.729 to 0.872)	(0.642 to 1.152)	(0.749 to 1.141)	(0.665 to 0.896)	(0.571 to 0.943)	(0.822 to 1.139)
Post 1	0.848**	0.890*	0.823***	0.850	0.967	0.775**	0.814	0.969
	(0.751 to 0.956)	(0.799 to 0.992)	(0.747 to 0.907)	(0.615 to 1.174)	(0.770 to 1.215)	(0.660 to 0.910)	(0.624 to 1.062)	(0.813 to 1.155)
Post 2	0.846*	0.813**	0.864**	0.875	0.936	0.773**	0.788	0.975
	(0.737 to 0.972)	(0.718 to 0.920)	(0.774 to 0.965)	(0.619 to 1.239)	(0.732 to 1.199)	(0.642 to 0.929)	(0.476 to 1.306)	(0.801 to 1.187)
Intervention	0.862***	0.898**	0.841***	0.840	0.916	0.834**	0.869	0.976
Overall*	(0.791 to 0.940)	(0.831 to 0.970)	(0.785 to 0.902)	(0.666 to 1.059)	(0.779 to 1.078)	(0.744 to 0.935)	(0.719 to 1.050)	(0.861 to 1.106)

*p<0·05, **p<0·01, ***p<0·001.

Parameters for each model in this table included terms for an intercept, linear change over time, seasonality (sine function) and an indicator for the evaluation period (preinvention as the reference category). Aggregate models (all sites and models for males and females) included separate intercept, slope and seasonality parameters for each site. Estimates of the effect of the intervention in table 2 are therefore adjusted for previous rates and trends within sites and seasonality.

* Combined effect of early, mid and late stage of intervention.

Earlymonths 1–8 of active LifeSpan implementationLatemonths 17–24 of LifeSpan implementationMidmonths 9–16 of LifeSpan implementation NSWNew South WalesPost 1months 1–8 following completion of the LifeSpan interventionPost 2months 9–16 following completion of the LifeSpan intervention, although length of final follow-up varies by sitePre5 years data prior to the transition phaseTransition6-month (10 at site 1) phase where sites planned for implementation and preceding active implementation of LifeSpan

There were substantial differences between sites, with the largest reduction coming from site 3 with a 16.6% decrease in ISH rates over the intervention period. Comparable but non-significant decreases of 16.0% and 13.1% over the full intervention period were observed in sites 1 and 4 respectively. Only the late stage of the intervention was statistically significant in site 4. The reduction in this site was consistent, but due to the intervention being implemented later than other sites, only a single month of second-stage postintervention data were available. Site 2 recorded a consistent but smaller, statistically non-significant decrease in rates (8.4% over the intervention stage; p=0.29).

Exploratory analyses revealed heterogeneity of effects by sex and between sites (table 2). For males, ISH reduced by 10.2% over the intervention period (IRR=0.90, 95% CI: 0.83 to 0.97, p=0∙01). The decrease in ISH in the early and mid-stages of the intervention for males was not significant; only in the late stage was a statistically significant reduction observed. The reduction was maintained after the intervention phase for the remainder of the postintervention period (table 2). For females, decreases in ISH were statistically significant at each stage of the intervention and afterwards, with a decrease over the intervention period of 14.8% (IRR=0.85, 95% CI: 0.78 to 0.90, p<0.001).

There were statistically non-significant changes in ISH rates for males and females in the rest of NSW during the intervention period (online supplemental table S1). However, a significant reduction of 13.8% (IRR=0.86, 95% CI: 0.80 to 0.93, p<0.001) was observed in the second postintervention period for males in NSW. The corresponding change in rates for females was a non-significant increase of 5.6% (IRR=1.06, 95% CI: 0.97 to 1.14, p=0.07).

### Suicide

**Figure 2:**
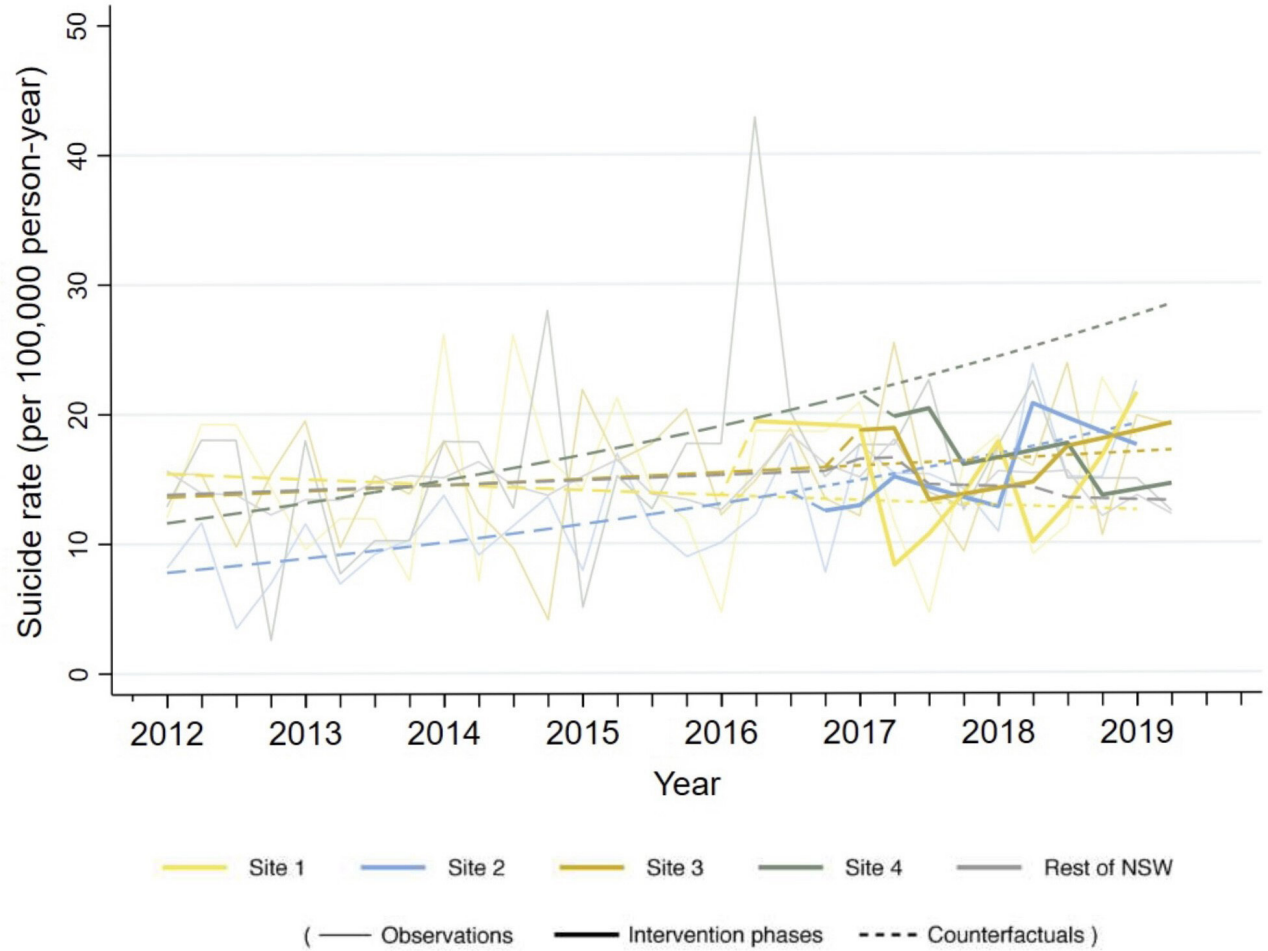
Changes over time in suicide rates (per 100 000) by trial sites and across New South Wales (NSW).

There were 625 suicide deaths identified during the preintervention period (table 1). The parameter estimates for the final model fitted across all sites. Corresponding observed and predicted suicide rates are graphed in figure 2. Over the total intervention period, LifeSpan was associated with a statistically non-significant decrease in suicide rates of 6.0% compared with preintervention (IRR=0.94, 95% CI: 0.74 to 1.18, p=0.582). Non-significant decreases in suicide rates were seen in the early and late implementation periods, and there were no differences in effects for suicide between sites at any stage of the trial, nor in NSW (see online supplemental table S2).

## Discussion

Implementation of LifeSpan was associated with a significant relative reduction in hospital-presenting ISH of 13.8% across all sites, and no statistically significant reductions were observed for suicide rates. The overall effect for ISH was most pronounced in the late stage of the intervention (18.5%) and appeared to be maintained for up to 8 months following the intervention period. No significant rate reductions in hospital-presenting ISH were observed for the rest of NSW during the study period. These findings suggest that LifeSpan could contribute to large decreases in the absolute numbers of self-harm cases in the general population.

Although we found positive changes in ISH rates overall, effects varied by site. Significant rate reductions were only observed in the overall implementation of site 3 and during the final implementation phase for site 4. While rate reductions in all sites were in the right direction, non-significant findings may be related to smaller IRRs and lower precision of the estimates, particularly in Site 1, and to the absence of important rate predictors (ie, sex, age) in the prespecified models.

In four communities in NSW where rates of suicide and/or ISH were higher than the state average, the LifeSpan multi-strategy intervention was observed to reduce rates of ISH at the population level. This finding may be attributable in part to our efforts to address methodological limitations of previous trials of multi-strategy suicide prevention interventions. It is the first study to use a pragmatic SW design to trial an intervention of this magnitude, providing ethical advantages over other trial designs,^22^ as all sites had equal opportunity to benefit from the LifeSpan intervention. Because it is one of the largest and most robust efforts to test the real-world impacts of whether prioritisation of evidence-based interventions reduces ISH rates at the population level, it has strong potential to inform policy and resourcing decisions to support actions that will improve suicide prevention efforts in the community. Rather than targeting ‘depression’ as per most previous multi-strategy models, for example,^4 5^ our model targeted behaviours and processes specific to suicide, based on evidence that such an approach has a greater likelihood of immediate effectiveness.^23^ The interventions were selected on the basis of demonstrating efficacy for outcomes relevant to this trial, to overcome issues in previous trials where there was over-prioritisation of strategies with limited-to-no intervention effects for suicide or self-harm, which likely contributed to the null effects.^8 9^ However, even in this trial, there was variation in the underlying effectiveness of the included strategies,^10^ which is the result of being constrained in the choices of programmes that have undergone rigorous testing.^24^ As more rigorously tested interventions become available in the suicide prevention field, the model should be revised to integrate current best practices, maximising its effectiveness potential.

Effect heterogeneity was observed between sites, similar to a prior Japanese trial.^6^ Significant reductions in self-harm rates were observed only in the final two sites to implement the model, and generally only in the late stage of implementation. The order of allocation may have contributed to this variation via the ‘learning effect’ mechanism. That is, late-starting sites likely benefitted from longer planning, access to all the intervention immediately post-transition and implementation learnings generated by earlier starting sites, enabling them to establish the lifespan model more quickly than sites 1 and 2. An insights paper on implementation characteristics and ingredients of success will be reported separately. Between-site differences are also likely to be partly attributable to differences in preimplementation ISH rates. Specifically, baseline ISH rates in site 2 were lower than all other sites, reducing the opportunity to effect significant change.

The post hoc inclusion of ‘sex’ as a constant term in our ISH models reduced error variance and resulted in changes in the effectiveness of the LifeSpan model. Although ISH rates significantly reduced for both sexes over the overall intervention period, effects were greater in females than males (15.9% vs 10.2%). For females, significant reductions in ISH were observed across all stages of the trial, whereas effects for males emerged in the late stage of intervention and then again in the second stage of postintervention follow-up. Because females have rates of ISH two to four times greater than males across the life course,^1^ the impacts of the LifeSpan model are important for this group. The sex variation in the effectiveness of LifeSpan may be partially explained by differences in patterns of service utilisation; females in suicidal distress are more likely to seek help than males,^25^ and therefore more likely to be exposed to interventions as well as being captured in administrative data.

### Limitations

Site 1 was delayed in crossing over, disrupting the intended structure of the trial by reducing the already low number of steps. This deviation from the original design may have reduced study power and affected our ability to disentangle intervention effects from time-related factors (eg, seasonality, policy changes). The SW design carries a risk of contamination relative to other randomised trial designs, and the later-starting sites may have had access to LifeSpan resources during the repeat baseline measure phase due to their involvement in preparing implementation guides. During the control period, all sites had some suicide prevention programmes in place to differing degrees, making it difficult to determine the causal effect of the intervention and leading to conservative effect estimates. Our expression of interest process may have introduced selection bias, whereby the chosen sites did not accurately represent health agencies across the state in terms of investment in suicide prevention. Baseline rates of self-harm varied between sites, and the sample size calculation was not adjusted to account for differences once sites were selected. As such, our study may be underpowered, resulting in failure to detect a reduction in ISH in the sites with the lowest rates. Moreover, the sample size was powered to ISH only and not change in suicide rates, which is an even rarer phenomenon. This limitation should be considered when interpreting the results, as our null effect for our suicide death endpoint may be an artefact of a lack of power rather than a true absence of effect. Future trials should power their sample size to detect change in rare endpoints to validate and expand on these findings. The intervention period of 2 years is short for a trial of this scale. It is likely to take time to see the full effects of the multi-strategy model, particularly for those interventions that have an upstream, protective function (eg, universal school-based interventions) relative to acute interventions for at-risk individuals. Our self-harm models did not account for repeated admissions, potentially violating the independence assumption and biasing our outcomes towards significance, even though the magnitude of the effects is likely to remain unbiased. Finally, the postintervention follow-up period varied between sites due to the SW design, and the final site only had 1 month of follow-up data in the post-two period. Therefore, the effects of the LifeSpan intervention on longer-term outcomes are not fully known.

## Conclusions

Delivering a complex suicide prevention model into health regions over a multiyear implementation period appears to be an effective approach to reducing rates of hospital-presenting self-harm in the population. Although reductions were modest, the implementation was brief for an intervention of this scale, and longer-term reductions could be expected.

## supplementary material

10.1136/bmjment-2024-301429 online supplemental file 1

## Data Availability

Data may be obtained from a third party and are not publicly available.
