# Supplementary material for: Effect of the LifeSpan suicide prevention model on self-harm and suicide in four communities in New South Wales, Australia: a stepped-wedge, cluster randomised controlled trial
Source: BMJ Ment Health. 2025 Mar 31;28(1):e301429. doi: 10.1136/bmjment-2024-301429 (PMC11962778; doi:10.1136/bmjment-2024-301429)
Supplement: online supplemental file 1 [file bmjment-28-1-s001.docx]

Supplementary materials

**S1 Text: LifeSpan strategy implementation targets**

| **Strategy** | **Description** | **Target** |
| --- | --- | --- |
| Establishment of Aftercare services in sites where such services do not exist, to provide support to individuals following a suicide attempt, and development and dissemination of best practice guidelines for effective crisis and aftercare for emergency departments (Improving emergency and follow-up care for suicidal crisis) | - Improved crisis care with new guidelines and training in EDs, education and resource packs distributed to individuals and families in crisis. Dedicated aftercare services for people who attempt suicide. - Better networks and information sharing between care providers and families. | - Review ED practices against crisis guidelines - Implement a dedicated aftercare service within the region |
| Training in psychosocial treatments for suicide to psychologists and allied health professionals (Using evidence-based treatment for suicidality) | - Delivering training in suicide prevention to clinicians plus guidelines for effective treatments including phone and web-based supports. - Improving information sharing between services, families and carers. - Developing preferred provider lists and improving local networks to close the gaps between primary care, allied health, schools and the hospital system. | - Train 22 per cent of the health professional workforce in evidence-based treatment |
| Capacity building and training in suicide detection for General Practitioners (Equipping primary care to identify and support people in distress) | - Delivering training in suicide prevention to GPs and practice staff and building better local care networks.   - Doctors to identify more patients in need using the StepCare program which:   - Screens all patients for depression, anxiety and suicidality   - Uses conversation starters to help GPs ask about suicide   - Auto-generates treatment plans, referrals (face to face and online therapies) and ongoing monitoring   - Integrates with existing programs such as Health Pathways. | - Implementation of a screening and response program (*StepCare*) into 9.5 per cent of general practices |
| Training for frontline responders (Improving the competency and confidence of frontline workers to deal with suicidal crisis) | - Local representatives from frontline services actively involved in helping improve the interactions those in suicidal crisis have with frontline staff. - Evidence-based training offered to frontline staff. | - No numeric targets set |
| School-based suicide prevention programs (Promoting help-seeking, mental health and resilience in schools) | - Deliver Youth Aware of Mental Health (YAM) program to Year 9 students in public schools; and working with headspace and others to deliver YAM in participating Independent and Catholic schools. - Providing Advanced Training in Suicide Prevention to school psychologists. - Training teachers to Question, Persuade and Refer (QPR) students who may be at risk of suicidal thinking. | 100% of public-school year nine classes to participate in the *Youth Aware of Mental Health* Program |
| Suicide Prevention Gatekeeper training (Training the community to recognise and response to suicidality) | - Local employers asked to provide QPR training to their staff. LifeSpan Champions help promote this at their workplace. - Offering QPR training free to the general public, and equipping those trained with referral information, resources, networks and support to maintain skills. | - Train one per cent of the adult population to recognise and respond to suicidality (gatekeeper training) |
| Public awareness raising of suicide and health promotion (Engaging the community and providing opportunities to be part of the change) | - - A local communication campaign to build awareness of how to help someone who may be suicidal and encourage people to undertake QPR (‘Question Persuade Refer’) training.   - Provide opportunities for community members, including those with lived experience of suicide, to get involved in local suicide prevention efforts. | - No numeric targets set |
| Training in best-practice MindFrame media reporting guidelines (Encouraging safe and purposeful media reporting) | - Taking a proactive, coordinated approach to working with the media and providing *Mindframe Plus* training to local media and organisations. - Developing a ‘Regional Suicide Response Plan’ to coordinate efforts, minimise media that may traumatise the community and increase coverage that promotes help seeking information. | - Conduct at least one *MindFrame Plus* training program for relevant stakeholders in each region - Establish a regional suicide response plan to suicides within each region |
| Means restriction activities (Improving safety and reducing access to means of suicide) | - Providing a regional Suicide Audit Report using coronial and hospital data, to support planning. - Working with local crisis services, Police, Ambulance, health services, pharmacies, suppliers, councils, media, politicians and others to identify and implement means safety. | - Suicide audit report delivered to regional suicide prevention collaboratives/alliances at baseline and 12 months, accompanied by means restriction reference manual |


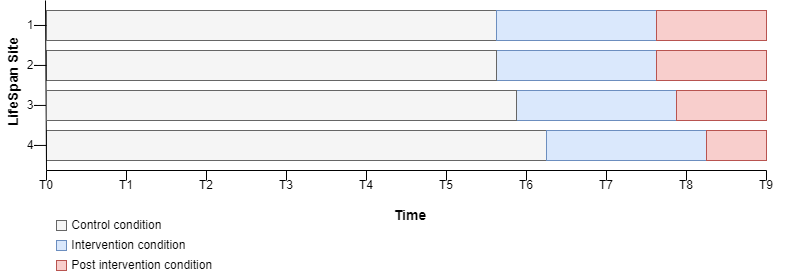


01 Aug 2017

31 Jul 2019

30 Nov 2019

01 Apr 2018

01 Dec 2017

31 Mar 2020

**Fig S1.** Overview of stepped-wedge trial design and measurement periods

Figure Footnotes: T0: 01 Jan 2012 – 31 Dec 2012; T1:01 Jan 2013 – 31 De 2013; T2: 01 Jan 2014 – 31 Dec 2014; T3: 01 Jan 2015 – 31 Dec 2015; T4: 01 Jan 2016 - 31 Dec 2016; T5: 01 Jan 2017 – 31 Dec 2017; T6: 01 Jan 2018 – 31 Dec 2018; T7: 01 Jan 2019 – 31 Dec 2019; T8: 01 Jan 2020 – 31 Dec 2020;

Control condition = non-active/pre-implementation period (no LifeSpan)

Intervention condition = 24-month active period of delivering LifeSpan;

Post intervention condition = each site ceased delivering LifeSpan (no LifeSpan) and outcomes monitored


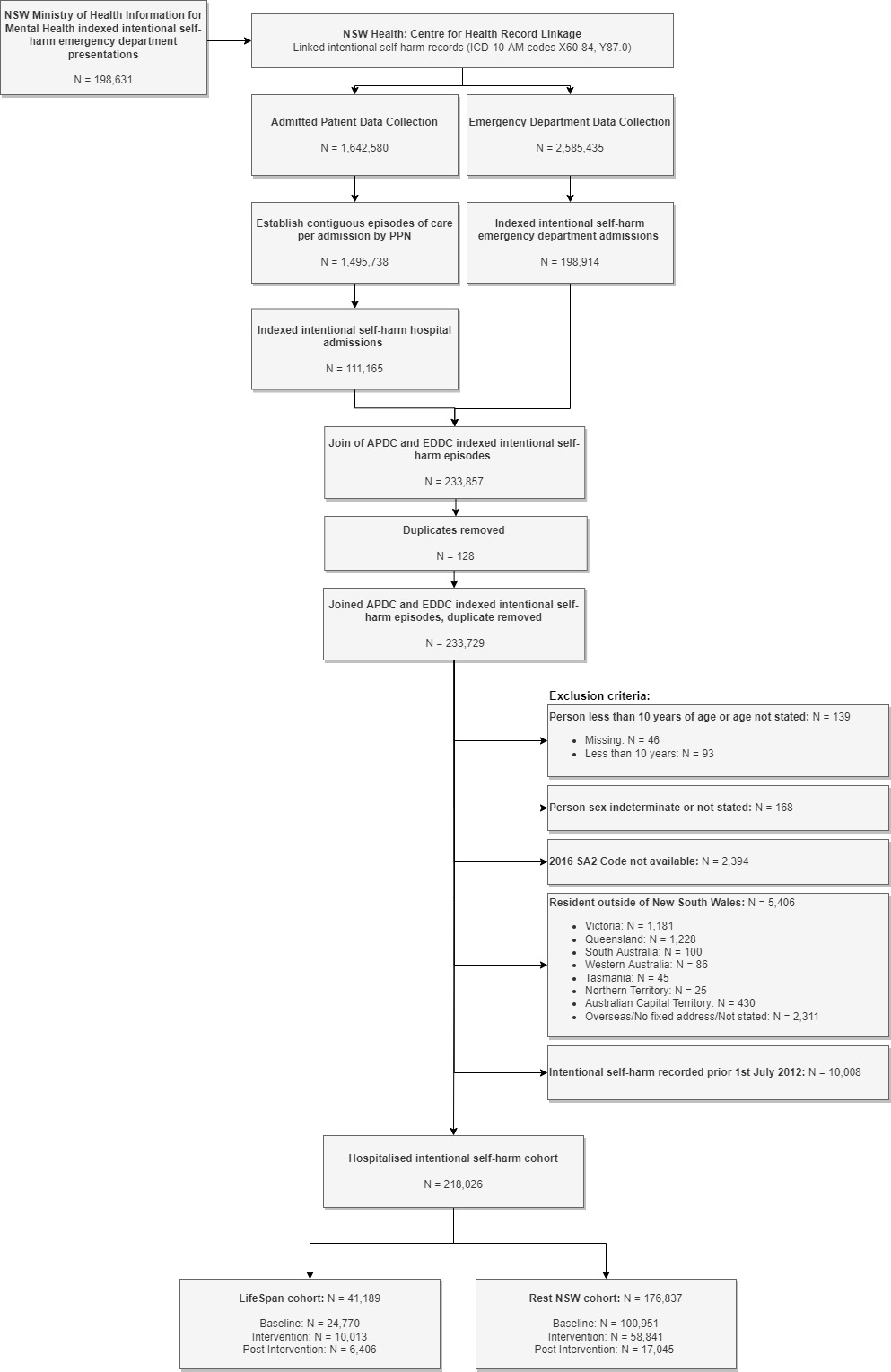


**Fig S2: number of records provided by the sampling frame**

**Table S1. Model parameter estimates expressed as incident rates (per 100,000) and incidence rate ratios for the model fitted to males and females separately in each site and to the rest of NSW (95% confidence intervals) for self-harm hospitalisations**

|  | **Site 1** | | **Site 2** | | **Site 3** | | **Site 4** | | **Rest of NSW** | |
| --- | --- | --- | --- | --- | --- | --- | --- | --- | --- | --- |
|  | **Male** | **Female** | **Male** | **Female** | **Male** | **Female** | **Male** | **Female** | **Male** | **Female** |
| Intercept | 33∙058**^***^** | 44∙799**^***^** | 26∙971**^***^** | 43∙466**^***^** | 28∙757**^***^** | 41∙513**^***^** | 29∙235**^***^** | 40∙887**^***^** | 21∙797**^***^** | 33∙911**^***^** |
|  | (29∙114-37∙538) | (40∙276-49∙830) | (24∙967-29∙135) | (40∙440-46∙720) | (26∙616-31∙072) | (38∙697-44∙534) | (26∙048-32∙812) | (37∙051-45∙119) | (21∙172-22∙441) | (32∙888-34∙966) |
| Slope | 1∙001 | 1∙005**^**^** | 1∙001 | 1∙001 | 1∙007**^***^** | 1∙005**^***^** | 1∙002 | 1∙007**^***^** | 1∙002**^***^** | 1∙001 |
|  | (0∙997-1∙005) | (1∙001-1∙008) | (0∙999-1∙004) | (0∙999-1∙003) | (1∙005-1∙009) | (1∙003-1∙007) | (0∙999-1∙005) | (1∙004-1∙009) | (1∙001-1∙003) | (1∙000-1∙002) |
| Month | 1∙059 | 1∙088**^**^** | 1∙095**^***^** | 1∙057**^**^** | 1∙057**^**^** | 1∙072**^***^** | 1∙104**^**^** | 1∙012 | 1∙068**^***^** | 1∙040**^***^** |
|  | (0∙992-1∙131) | (1∙032-1∙147) | (1∙051-1∙141) | (1∙017-1∙099) | (1∙017-1∙099) | (1∙034-1∙113) | (1∙034-1∙179) | (0∙959-1∙069) | (1∙051-1∙084) | (1∙022-1∙057) |
|  |  |  |  |  |  |  |  |  |  |  |
| Pre | 1 | 1 | 1 | 1 | 1 | 1 | 1 | 1 | 1 | 1 |
|  | — | — | — | — | — | — | — | — | — | — |
| Transition | 0∙837 | 0∙856 | 0∙883 | 0∙981 | 0∙936 | 0∙827**^**^** | 0∙976 | 0∙886 | 0∙954 | 1∙033 |
|  | (0∙682-1∙027) | (0∙727-1∙008) | (0∙762-1∙022) | (0∙858-1∙120) | (0∙824-1∙064) | (0∙728-0∙939) | (0∙787-1∙211) | (0∙741-1∙058) | (0∙906-1∙004) | (0∙978-1∙091) |
| Early | 0∙793 | 0∙805**^*^** | 0∙895 | 0∙943 | 0∙888 | 0∙824**^**^** | 1∙206 | 0∙961 | 0∙972 | 0∙979 |
|  | (0∙620-1∙013) | (0∙662-0∙978) | (0∙780-1∙026) | (0∙829-1∙073) | (0∙783-1∙006) | (0∙729-0∙931) | (0∙989-1∙472) | (0∙815-1∙133) | (0∙923-1∙024) | (0∙926-1∙036) |
| Mid | 0∙918 | 0∙822 | 0∙949 | 0∙874 | 0∙915 | 0∙854**^*^** | 0∙907 | 0∙828**^*^** | 0∙969 | 0∙993 |
|  | (0∙706-1∙193) | (0∙664-1∙017) | (0∙817-1∙103) | (0∙757-1∙010) | (0∙800-1∙046) | (0∙749-0∙973) | (0∙725-1∙135) | (0∙691-0∙991) | (0∙916-1∙025) | (0∙935-1∙056) |
| Late | 0∙894 | 0∙835 | 0∙977 | 0∙89 | 0∙762**^***^** | 0∙779**^***^** | 0∙785 | 0∙705**^***^** | 0∙953 | 0∙977 |
|  | (0∙670-1∙192) | (0∙661-1∙055) | (0∙832-1∙148) | (0∙762-1∙040) | (0∙655-0∙886) | (0∙675-0∙900) | (0∙613-1∙006) | (0∙577-0∙862) | (0∙895-1∙014) | (0∙913-1∙044) |
| Post 1 | 0∙914 | 0∙809 | 1∙003 | 0∙947 | 0∙758**^***^** | 0∙787**^**^** | 0∙973 | 0∙735**^**^** | 0∙955 | 0∙978 |
|  | (0∙665-1∙257) | (0∙625-1∙048) | (0∙844-1∙194) | (0∙801-1∙119) | (0∙645-0∙891) | (0∙675-0∙918) | (0∙752-1∙259) | (0∙595-0∙908) | (0∙893-1∙021) | (0∙910-1∙051) |
| Post 2 | 0∙808 | 0∙898 | 0∙912 | 0∙95 | 0∙711**^***^** | 0∙820**^*^** | 0∙862 | 0∙748 | 0∙862**^***^** | 1∙056 |
|  | (0∙572-1∙141) | (0∙680-1∙185) | (0∙753-1∙105) | (0∙793-1∙139) | (0∙590-0∙856) | (0∙688-0∙978) | (0∙531-1∙400) | (0∙505-1∙110) | (0∙800-0∙930) | (0∙975-1∙144) |
| Intervention overall† | 0∙866 | 0∙820**^*^** | 0∙940 | 0∙902 | 0∙852**^***^** | 0∙818**^***^** | 0∙951 | 0∙825**^*^** | 0∙964 | 0∙983 |
|  | (0∙689-1∙089) | (0∙681-0∙988) | (0∙829-1∙065) | (0∙801-1∙017) | (0∙760-0∙956) | (0∙733-0∙913) | (0∙791-1∙143) | (0∙710-0∙958) | (0∙919-1∙012) | (0∙934-1∙035) |

† Combined effect of early**-** mid and late stage of intervention; **^*^** p<0∙05**,** **^**^** p<0∙01**,** **^***^** p<0∙001; Pre = five years prior to the transition phase; Transition = 6-month (10 at site 1) phase where sites planned for implementation and preceding active implementation of LifeSpan; Early = months 1 – 8 of active LifeSpan implementation; Mid = months 9–16 of LifeSpan implementation; Late = months 17–24 of LifeSpan implementation; Post 1 = months 1–8 following completion of the LifeSpan intervention; Post 2 = months 9 – 16 following completion of the LifeSpan intervention**–** although length of final follow up varies by site)

**Table S2. Suicide deaths model parameter estimates expressed as incident rates (per 100,000 person-years) and incidence rate ratios for the model fitted to all sites, to each site separately, and the rest of New South Wales (95% confidence intervals)**

|  | **All sites** | **Individual Site** | | | | **Rest of NSW** |
| --- | --- | --- | --- | --- | --- | --- |
|  |  | **Site 1** | **Site 2** | **Site 3** | **Site 4** |  |
| **Intercept** | | | | | | |
| Site 1 | 14.198*** | 15.5358*** | — | — | — | — |
|  | (10.358-19.461) | (10.4684-23.0564) |  |  |  |  |
| Site 2 | 7.359*** | — | 7.557*** | — | — | — |
|  | (5.539-9.778) |  | (5.410-10.556) |  |  |  |
| Site 3 | 12.978*** | — | — | 13.501 *** | — | — |
|  | (10.100-16.678) |  |  | (10.149-17.960) |  |  |
| Site 4 | 13.246 | — | — | — | 11.268*** | — |
|  | (9.618-18.244) |  |  |  | (7.660-16.575) |  |
| Rest of NSW | — | — | — | — | — | 13.719*** |
|  |  |  |  |  |  | (12.593-14.944) |
| **Slope** | | | | | | |
| Site 1 | 1.005 | 1.300* | — | — | — |  |
|  | (0.984-1.027) | (1.002-1.687) |  |  |  |  |
| Site 2 | 1.035 | — | 0.916 | — | — | — |
|  | (1.016-1.055) |  | (0.779-1.076) |  |  |  |
| Site 3 | 1.012 | — | — | 1.024 | — | — |
|  | (0.995-1.030) |  |  | (0.856-1.226) |  |  |
| Site 4 | 1.015 | — | — | — | 1.001 | — |
|  | (0.995-1.035) |  |  |  | (0.746-1.344) |  |
| Rest of NSW | — | — | — | — | — | 0.988 |
|  |  |  |  |  |  | (0.933-1.047) |
| **Evaluation period** | | | | | | |
| Pre | 1 | 1 | 1 | 1 | 1 | 1 |
|  | — | — | — | — | — | — |
| Transition | 1.063 | 1.426 | 0.868 | 1.171 | 0.889 | 1.053 |
|  | (0.823-1.373) | (0.807-2.521) | (0.523-1.438) | (0.735-1.865) | (0.489-1.617) | (0.907-1.221) |
| Early | 0.783 | 0.482 | 1.076 | 0.801 | 0.678 | 0.927 |
|  | (0.552-1.111) | (0.181-1.286) | (0.611-1.894) | (0.432-1.484) | (0.274-1.680) | (0.765-1.122) |
| Late | 0.832 | 0.211 | 1.848 | 0.921 | 0.508 | 0.879 |
|  | (0.417-1.657) | (0.033-1.365) | (0.619-5.511) | (0.270-3.142) | (0.079-3.266) | (0.595-1.297) |
| Intervention overall^†^ | 0.937 | 0.519 | 0.945 | 0.720 | 0.904 | 0.928 |
|  | (0.744-1.180) | (0.245-1.101) | (0.585-1.053) | (0.428-1.211) | (0.510-1.606) | (0.791-1.089) |
